# A Novel Recombinant Chicken-Derived H6N8 Subtype Avian Influenza Virus Caused Disease in Chickens and Mice

**DOI:** 10.3390/v18010012

**Published:** 2025-12-21

**Authors:** Yafen Song, Shengyao Song, Aoyang Yan, Hongxuan Gong, Huan Yang, Feihu Guan, Min Zhang, Bing Zhang, Qianyi Zhang, Chenghuai Yang, Guanlong Xu

**Affiliations:** 1 China Institute of Veterinary Drug Control, Beijing 100081, China; songyafen1@126.com (Y.S.); gfh9826@163.com (F.G.);; 2 College of Veterinary Medicine, Qingdao Agricultural University, Qingdao 266109, China; 3 College of Animal Science and Technology, Shihezi University, Shihezi 832000, China

**Keywords:** H6N8, avian influenza virus, triple-reassortant, phylogenetic analysis, pathogenicity, antibody

## Abstract

H6 subtype avian influenza viruses (AIVs) have a broader host range and circulate globally in wild birds, domestic ducks, geese, and terrestrial poultry all over the world. Their demonstrated capacity to adapt receptor-binding affinity for mammalian species constitutes a persistent concern for zoonotic transmission and public health. In this study, a novel triple-reassortant H6N8 AIV strain was isolated from a chicken farm in southern China and designated as A/chicken/Guangdong/JM642/2023 (H6N8). The complete genome of the virus was sequenced using Next-Generation Sequencing. Phylogenetic analysis indicated that the HA gene of the isolate clustered into the Group III/HN573-like, which encompasses H6 subtype viruses bearing various NA genes and belonging to the Eurasian gene pool. The NA gene showed the closest genetic relationship with viruses originating from North America. All six internal genes were derived from H9N2 AIVs. The virus possesses several key molecular determinants known to contribute to an expanded host range and increased virulence. Animal infection studies demonstrated that the virus was capable of infecting mice without prior adaptation. It replicated efficiently in the lungs and nasal turbinates, followed by systemic dissemination resulting in lethal outcomes. Inoculated chickens remained asymptomatic; however, the virus replicated efficiently in multiple organs, with high viral loads detected particularly in the lungs and kidneys. Viral shedding occurred via both the respiratory and digestive tracts, and horizontal transmission was observed among chickens. Notably, infected and contacted chickens developed high levels of antibodies from 8 days post-inoculation (DPI) to the end of observation period. This study enhances our understanding of the genetic and biological characteristics of the novel reassortant H6N8 AIVs and underscores their potential risk to public health.

## 1. Introduction

AIVs not only cause huge economic losses to the poultry industry but also continuously present a challenge to public health. Research and pandemic preparedness have largely focused on the H5, H7, and H9 subtype influenza viruses. However, there has been relatively little genetic and biologic characterization of the H6 viruses. Since the first avian influenza virus of the H6 subtype was detected from a turkey in the United States in 1965, H6 viruses have become an increasingly persistent burden for the poultry industry and frequently introduce large-scale disease [1]. The viruses have been transmitted with frequency from avian species in their natural habitats to domesticated poultry populations and are known to be widely distributed among diverse avian species including migratory waterfowl and domestic aquatic and terrestrial avian species throughout the world [2]. In addition to harming wild birds and poultry, studies have revealed that the H6 viruses obtained several mammalian adaptation motifs indicative of mammalian adaptation and had the ability to cross the species barrier and directly infect mammals. Studies demonstrated that mice and ferrets have been infected with the H6 viruses without adaption [3]. Pigs infected with the H6N6 virus were found in Guangdong province, China, in 2010 [4]. The first natural avian-origin H6N1 influenza A virus infection case in dogs was confirmed in Taiwan in 2014 [5]. The first human infected with the H6N1 virus was reported in Taiwan in 2013 [6].

H6 viruses likely play an important role in the ecology of influenza A viruses through reassortment with other AIVs. Such reassortments may change the biologic characterization of the virus. H6 viruses have been reported to provide internal genes for other subtype viruses and generate novel viruses frequently, such as H5N1, H5N6, and H9N2 AIVs, which were detected in humans [7]. Thus, it is very important to strengthen the epidemiological investigation of the H6 subtype AIVs.

Surveillance data have revealed that the predominant subtypes of H6 viruses were H6N1 and H6N2 prior to 2005. Since 2006, the isolation rate of the H6N6 subtype AIVs has begun to increase [2]. Although the number of H6N8 viruses has remained relatively stable for decades, comprehensive understanding of their genetic and biological characteristics remains limited. Here, we determined the genetic evolution of a novel H6N8 isolate and characterized the replication and pathogenicity ability in both chickens and mice. Our results revealed the potential threat to public health posed by the novel reassortant H6N8 AIV isolated from chickens.

## 2. Materials and Methods

### 2.1. Eggs, Chickens, and BALB/c Mice

Five-week-old specific-pathogen-free (SPF) female BALB/c mice were purchased from Beijing Vital River Laboratory Animal Technology Co., Ltd., Beijing, China. The 6-week-old white leghorn SPF chickens and 9–10-day-old SPF embryonated chicken eggs were purchased from Beijing Boehringer Ingelheim Vital Biotechnology Co., Ltd., Beijing, China.

### 2.2. Virus Isolation and Identification

The H6N8 virus, A/chicken/Guangdong/JM642/2023 (H6N8) (JM642), was isolated from mixed oropharyngeal and cloacal samples of apparently healthy chickens during routine surveillance activities in chicken farms in Guangdong Province in the winter of 2023. Briefly, the mixed oropharyngeal and cloacal sample was inoculated into the allantoic cavity of 9-day-old SPF embryonated chicken eggs. After incubation at 37 °C for 96 h, the allantoic fluid was harvested for testing. The virus was identified by a hemagglutination test, reverse-transcription polymerase-chain reaction (RT-PCR), and a hemagglutination inhibition (HI) test. The isolate was subsequently passaged three times by limiting the dilution assay before deep-sequencing analysis and animal study. Values of 50% embryo infective doses (EID_50_) and 50% egg lethal doses (ELD_50_) were calculated by the Reed–Muench method [8].

### 2.3. Genetic Sequencing and Analysis

The viral RNA was extracted from the allantoic fluid of a chicken embryo using TaKaRa MiniBEST Viral RNA/DNA Extraction Kit Ver.5.0 (TaKaRa, Beijing, China) and prepared for next-generation sequencing. Illumina sequencing, library construction, and gene sequencing data analysis were performed at the Beijing Tsingke Biotechnology Co., Ltd. (Beijing, China). The representative viruses were downloaded from Genbank and GISAID EpiFlu™ database. All sequences were assembled, edited, and aligned, and residue analysis was performed using Lasergene 7.1. Phylogenetic trees were generated by the distance-based neighbor-joining method, using MEGA 4.0 software. The reliability of the trees was assessed by bootstrap analysis with 1000 replicates. Horizontal distances were proportional to the genetic distance. The nucleotide sequences are available from GenBank under the accession numbers: PX495069-PX495076.

### 2.4. Animal Experiments [9]

#### 2.4.1. Ethical Compliance

All experiments were conducted in biosafety level 2 (BSL2) laboratories. The handling of chickens and mice were performed in accordance with the approved guidelines of the Experimental Animal Administration and Ethics Committee of the 106 China Institute of Veterinary Drug Control [Permit Number 202500266; 24 July 2025].

#### 2.4.2. Infection Experiment in SPF Chickens

Twenty-five six 6-week-old white leghorn SPF chickens were intranasally inoculated with 10^6.0^ EID_50_ of the JM642 virus in 0.2 mL. At 24 h post-infection, five chickens were inoculated intranasally with 0.2 mL PBS as a contact group, housed with those chickens inoculated with the JM642 virus. All chickens were observed for clinical signs for 14 days. Oropharyngeal and cloacal swabs were collected on 1, 3, 5, 7, 9, 11, and 13 DPI. In order to detect the virus replication, three infected chickens were killed on 3 and 5 DPI, and their hearts, livers, spleens, lungs, kidneys, brains, duodena, pancreases, and trachea were collected. All swabs were processed in accordance with reference [9] (primers used in this study, H6-forword: 5′-ATGATTGCAWTCATTGTAATAGC-3′; H6-reverse: 5′-TCATATACATATTCTGCACTG-3′), and all tissues were titrated for virus titers in eggs [9]. Blood samples were collected on 3, 5, 8, 11, and 14 DPI from infected and contacted chickens, and the serum samples were then used to test the antibody response using the HI test.

#### 2.4.3. Infection Experiment in BALB/c Mice

To evaluate the virulence of the virus, groups of five BALB/c mice were anesthetized with CO_2_ and inoculated intranasally with 10-fold serial dilutions of the virus, and five mice were inoculated with PBS as the control. The mice were monitored daily for weight loss and mortality for 14 days. Mice that showed severe symptoms or lost more than 25% of their body weight were euthanized and scored as dead for humane reasons. The MLD_50_ value was calculated according to the method of Reed and Muench.

To evaluate the viral replication in mice, six mice were inoculated intranasally with 10^6.0^ EID_50_ of the JM642 virus in a volume of 0.05 mL, and six mice were inoculated with PBS as the control. Three mice in each group were euthanized on 3 and 5 DPI, and their hearts, livers, spleens, lungs, kidneys, and brains were collected for virus titration in eggs.

### 2.5. Statistical Analysis

Statistical analyses were performed with a two-way ANOVA test using GraphPad Prism 7.0 software (GraphPad Software Inc., San Diego, CA, USA), A *p*-value < 0.05 was considered to be statistically significant (* *p* < 0.05; ** *p* < 0.01).

## 3. Results

### 3.1. Phylogenic Analysis and Molecular Characterization

**Figure 1:**
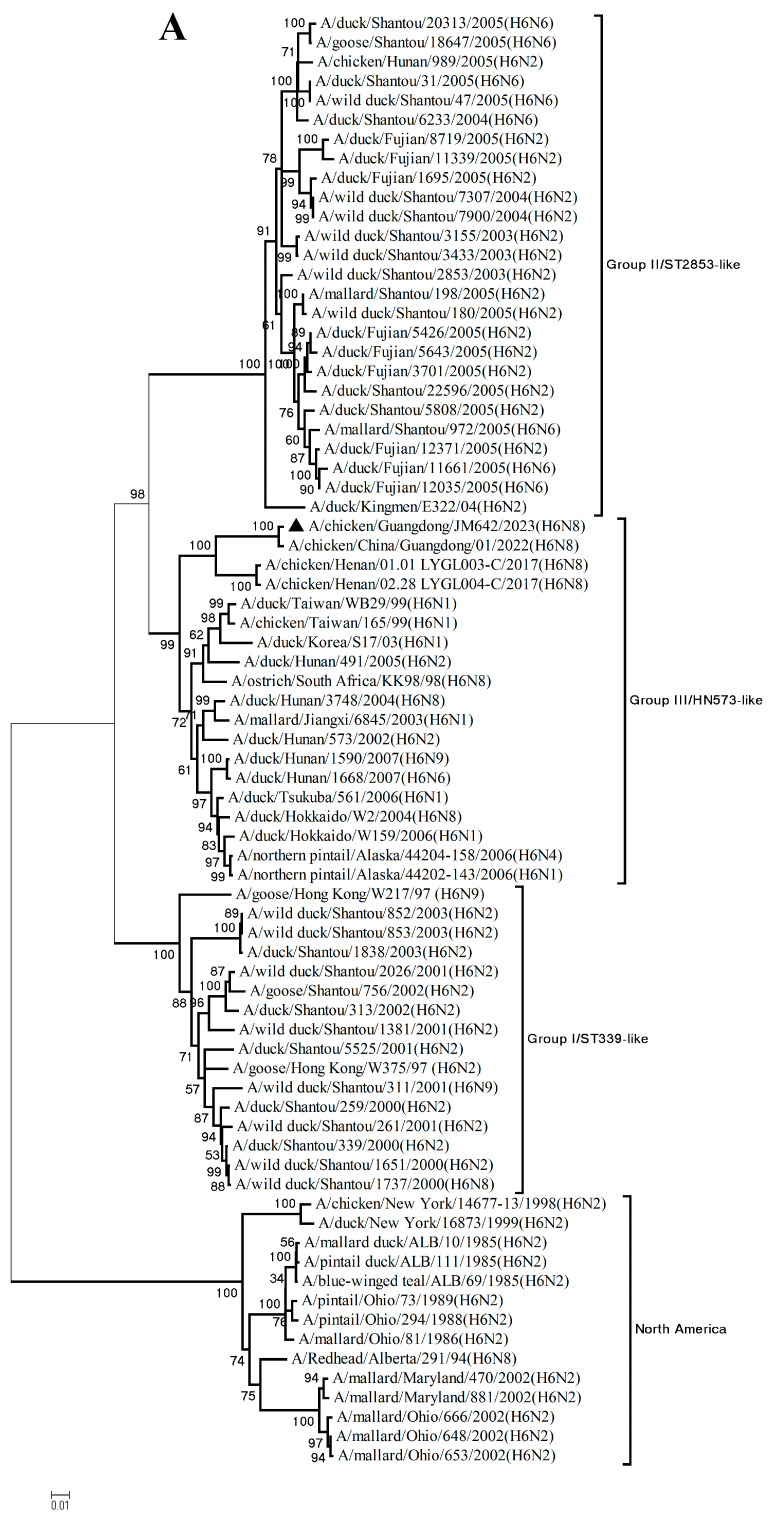

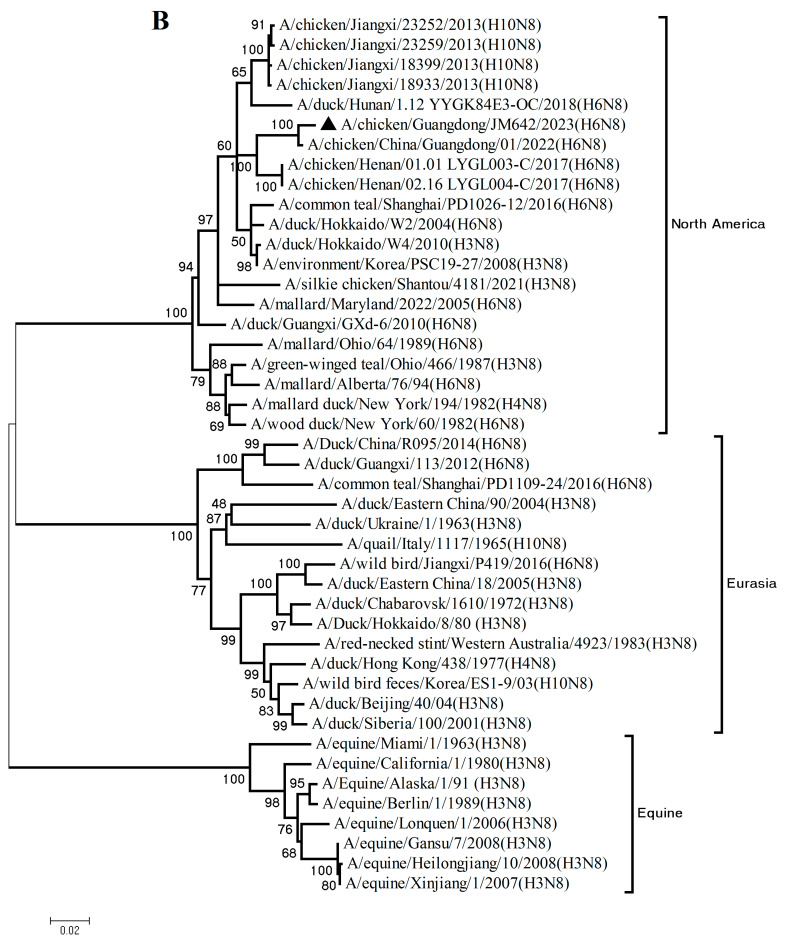

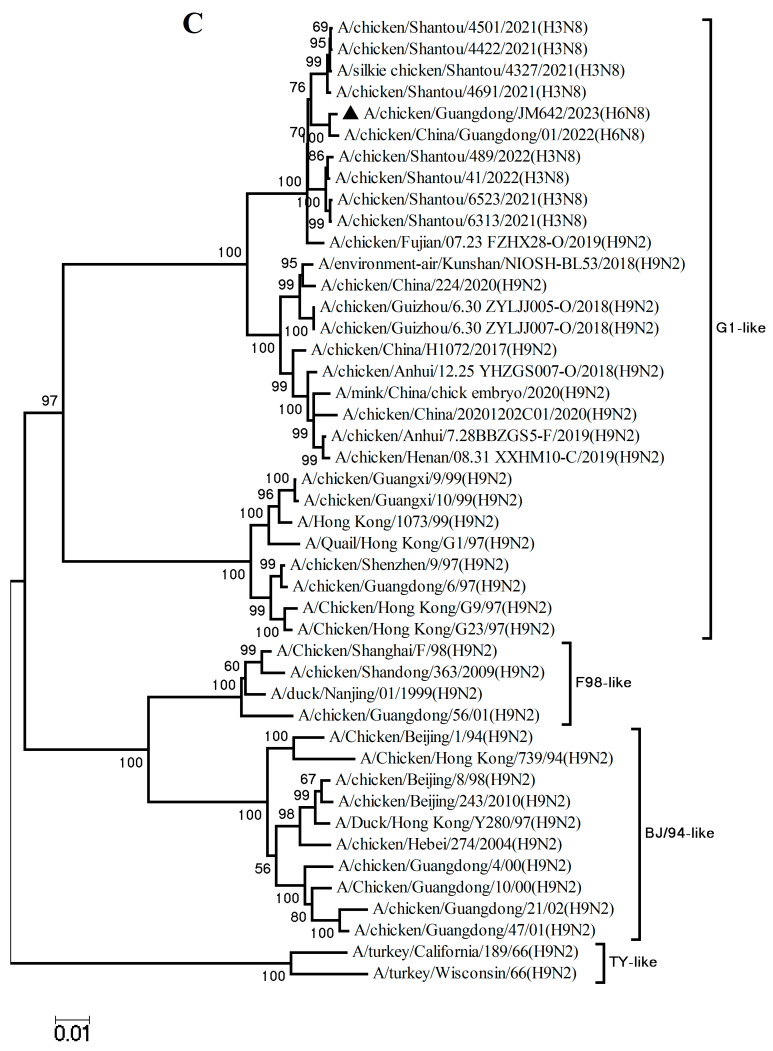

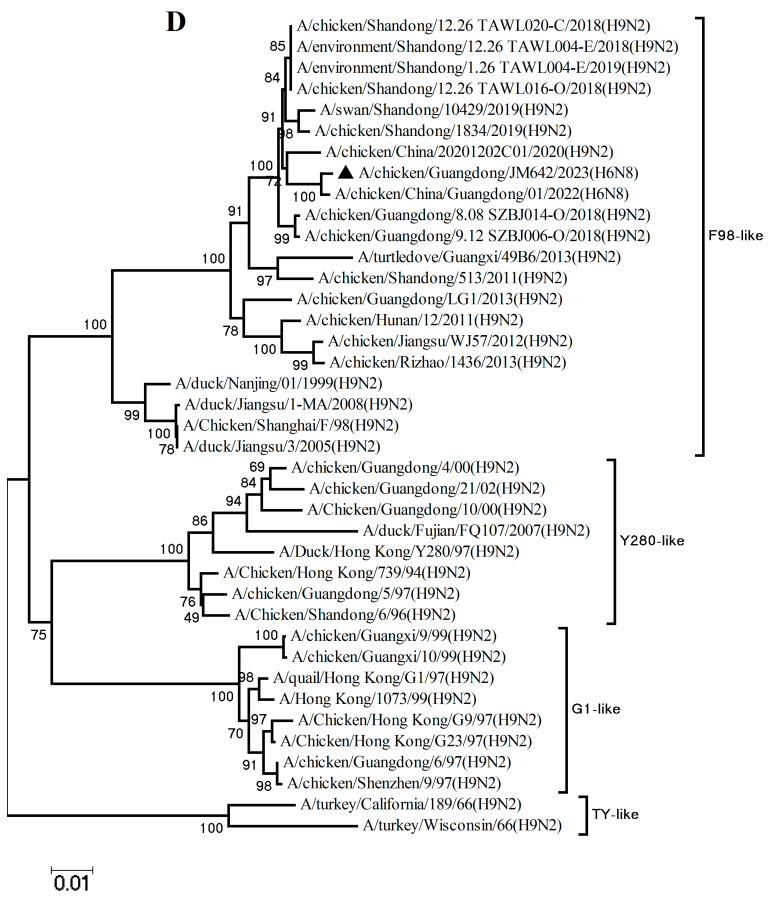

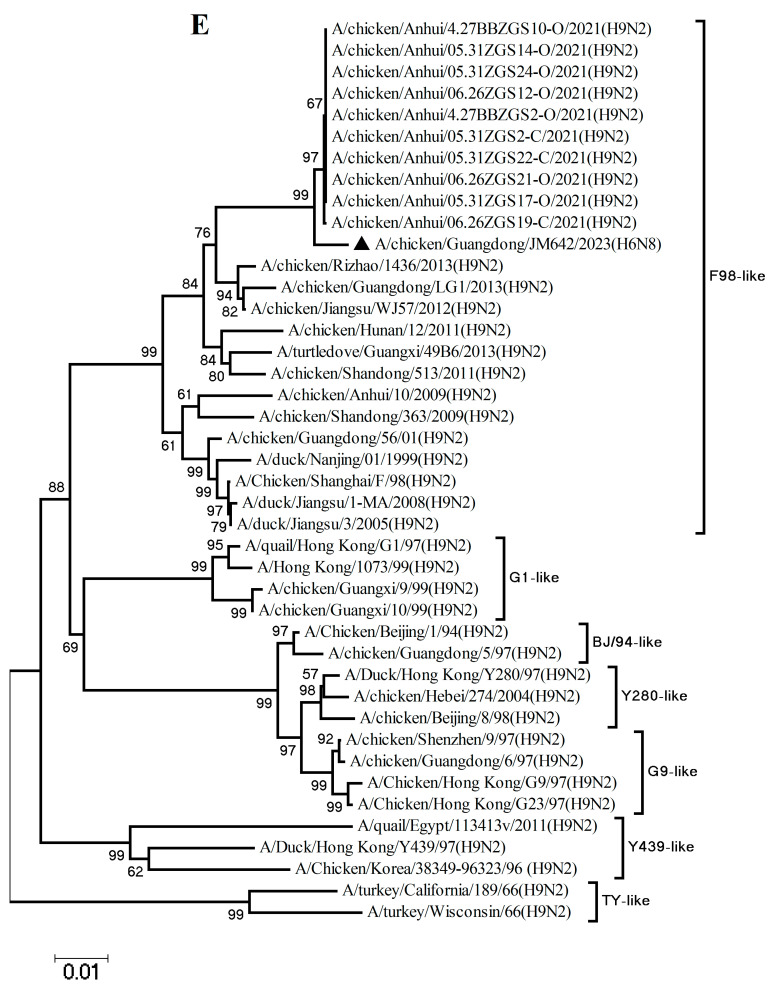

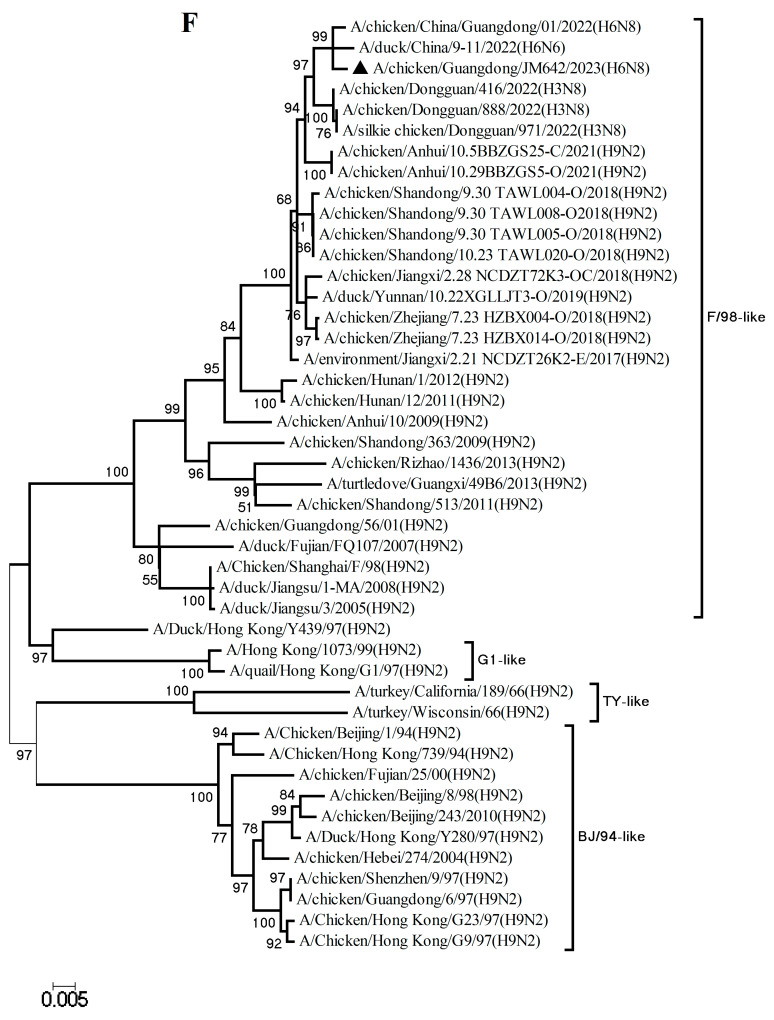

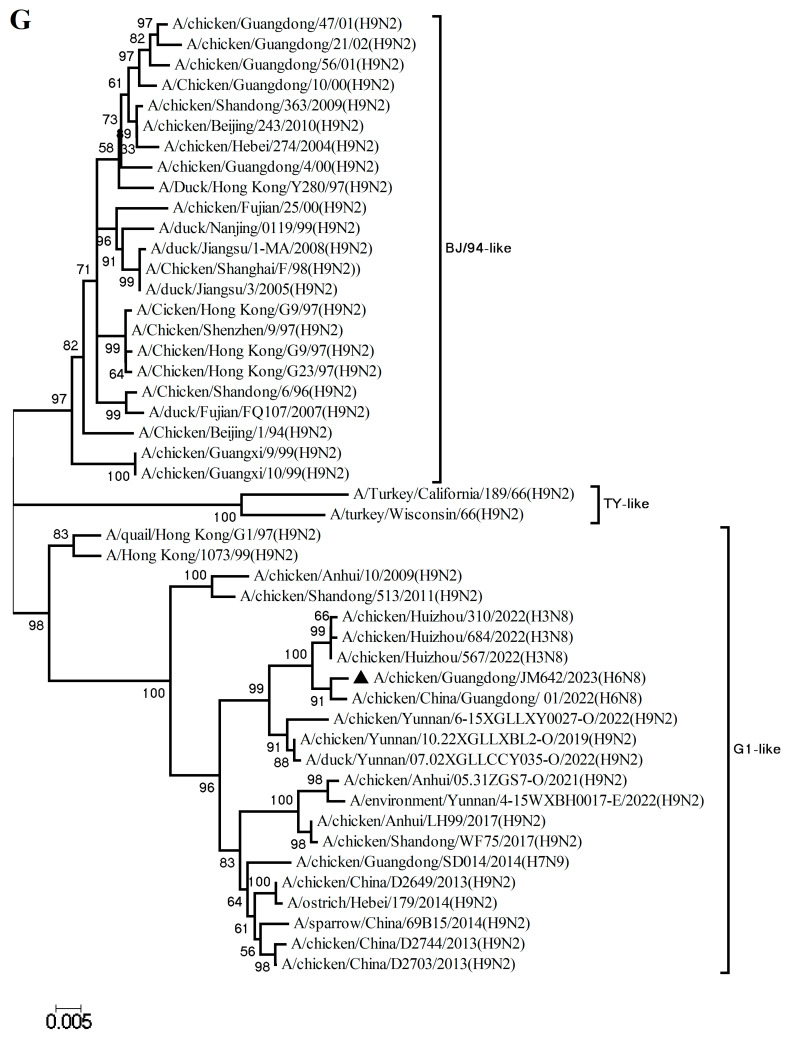

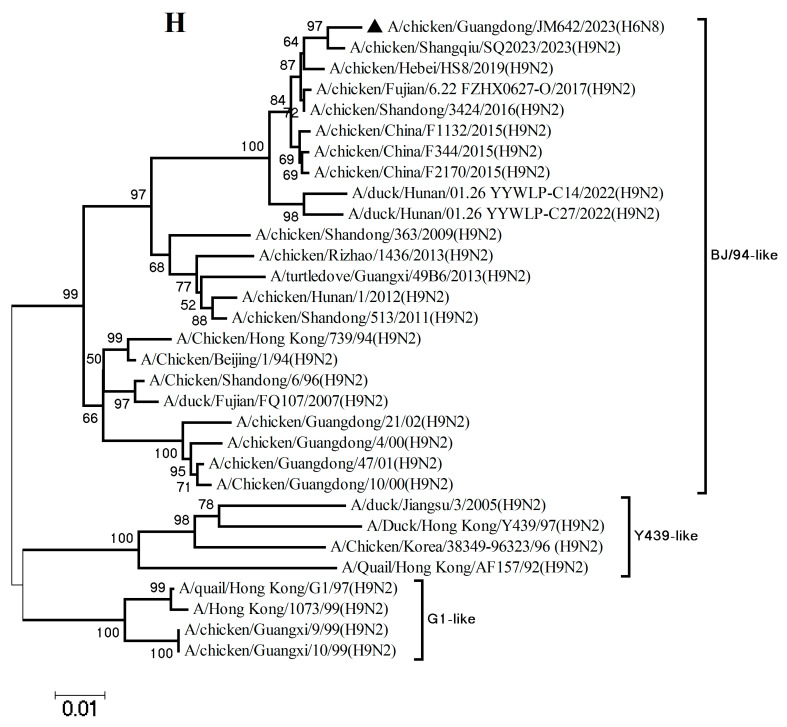
Phylogenetic trees of the (**A**) hemagglutinin (HA), (**B**) neuraminidase (NA), (**C**) polymerase basic subunit 2 (PB2), (**D**) polymerase basic subunit 1 (PB1), (**E**) polymerase acidic subunit (PA), (**F**) nucleoprotein (NP), (**G**) matrix (M), and (**H**) nonstructural (NS) genes of the JM642 virus. The virus in this study was marked with a black triangle “▲”.

Phylogenetic analysis revealed that the HA gene fell into the Group III/HN573-like lineage represented by A/duck/Hunan/573/2002(H6N2) (or HN573-like) [10], which encompasses H6 subtype viruses bearing various NA genes, including N1, N2, N4, N5, N6, N8, and N9 (Figure 1A). Viruses of this lineage obviously belong to the Eurasian gene pool, which are phylogenetically related to H6 viruses isolated from wild or migratory birds and include some viruses isolated from poultry disease outbreaks from different countries and regions. The NA gene fell into the North America lineage, which included viruses originating from poultry, waterfowl, and wild birds (Figure 1B). The six internal genes of the isolate were derived from H9N2 AIVs [11]. The PB2 and M genes belonged to the G1-like lineage represented by A/quail/Hong Kong/G1/97 (H9N2) (Figure 1C,G). The PB1, PA, and NP genes fell into the F98-like lineage represented by A/chicken/Shanghai/F/98 (H9N2) (Figure 1D–F). The NS gene fell into the BJ/94-like lineage represented by A/chicken/Beijing/1/94 (H9N2) (Figure 1H).

We analyzed the key amino acid (aa) sites related to the host range, pathogenicity, and drug resistance of the JM642 virus (Table 1 and Table 2). The results showed that the HA gene possessed the sequence PQIETR↓G at the cleavage site between HA1 and HA2, which conformed to the characteristics of low-pathogenicity AIVs [12]. The amino acid residues in positions 226 and 228 (H3 numbering) of the virus were Q226 and G228, respectively, which suggested that the virus retained a strong preference for an avian receptor [13]. However, the presence of mutations T155I, G158N, T160A, and E190V within the receptor-binding site of HA is predicted to confer a slight preference for human-type receptors [14,15,16]. PB2-E627K and PB2-D701N substitution were not detected in the virus, which are the major markers for the host range and the virulence of influenza viruses. However, PB2-G158E, PB2-I292V, PB2-A588V, PB1-M317V, PB1-P598L, PA-A515T, NA-64~77 aa delete, M1-N30D, and NS1-P42S were detected in the virus, which are known to critically shape the replication, virulence, and transmissibility of influenza viruses [2,17,18,19,20]. The drug resistance sites in NA-H274Y and NA-R292K substitution were not found in the virus [21], but M-S31N substitution was detected, which indicated that the JM642 virus has a reduced susceptibility to amantadine and rimantadine [22,23,24].

### 3.2. Pathogenicity of the Virus to Chickens

**Figure 2:**
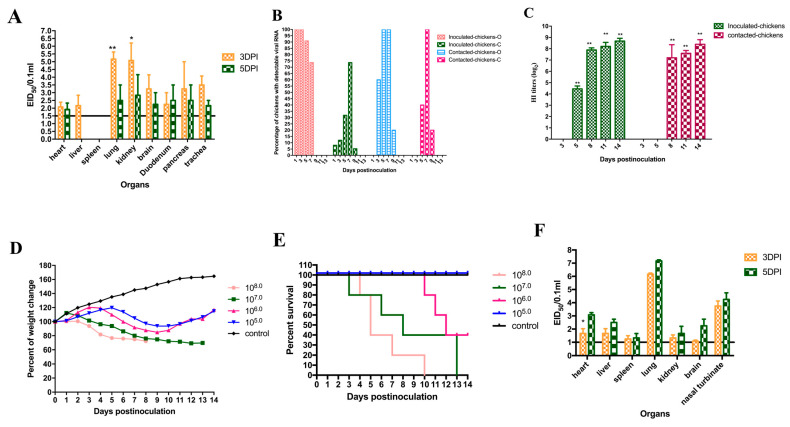
Pathogenicity and transmission of the JM642 virus in chickens and mice. SPF chickens and BALB/c mice were inoculated intranasally with 10^6^ EID_50_ of the JM642 virus at 0.2 mL and 0.05 mL in volume, respectively. Three chickens and mice were killed on 3 and 5 DPI, respectively. Organs were collected for virus titration in eggs. The virus titers were shown as means ± SD. A *p*-value < 0.05 was considered to be statistically significant (* *p* < 0.05; ** *p* < 0.01). (**A**,**F**) Replication in the chickens and mice of the JM642 virus on 3 and 5 DPI. (**B**) Virus shedding from the inoculated and contacted chickens. Inoculated chickens-O represents virus shedding from oropharyngeal swabs of the inoculated chickens, inoculated chickens-C represents virus shedding from cloacal swabs of the inoculated chickens, contacted chickens-O represents virus shedding from oropharyngeal swabs of the contacted chickens, and contacted chickens-C represents virus shedding from cloacal swabs of the contacted chickens. (**C**) HI antibody titers of the inoculated and contacted chickens during the observation period (**D**,**E**) Weight change and survival rates of BABL/c mice during the 14 DPI.

The JM642 virus, isolated from apparently healthy chickens, grew efficiently in eggs with virus titers of 10^8.50^ EID_50_/0.1 mL and 10^8.00^ ELD_50_/0.1 mL. Chickens inoculated with 10^6.0^ EID_50_ of the virus did not show significant clinical syndromes, and all survived through the end of the experiment. The JM642 virus could be detected in multiple organs of the inoculated chickens on 3 DPI and 5 DPI (Figure 2A). At 3 DPI, the virus could replicate efficiently in the lungs and kidneys, with mean titers of 5.17 ± 0.80 and 5.08 ± 1.94 EID_50_/0.1 mL, respectively. Compared to the lungs and kidneys, lower viral titers were detected in the hearts, livers, brains, duodena, pancreases, and trachea; their mean titers were 2.08 ± 0.52, 2.17 ± 1.16, 3.25 ± 1.56, 2.25 ± 1.30, 3.25 ± 3.03, and 3.50 ± 1.00 log_10_EID_50_/0.1 mL, respectively. The virus could not be detected in spleens. At 5 DPI, compared to 3 DPI, the virus replicated poorly in the hearts, lungs, kidneys, brains, duodena, pancreases, and trachea, and their mean titers were 1.92 ± 0.72, 2.50 ± 1.73 (*p* < 0.01), 2.83 ± 2.31 (*p* < 0.01), 2.25 ± 1.30, 2.50 ± 1.73, 2.50 ± 1.73, and 2.17 ± 0.58 log_10_EID_50_/0.1 mL, respectively. The virus could not be detected in livers and spleens.

Oropharyngeal and cloacal swabs from the chickens inoculated with the JM642 virus were obtained to measure the viral shedding. The results showed that the viral shedding was detected from oropharyngeal swabs of all the inoculated chickens at 1 and 3 DPI, and 90.90% and 73.68% chickens shed the virus at 5 and 7 DPI, respectively. No viral shedding from oropharyngeal swabs of the inoculated chickens was detected at 9, 11, and 13 DPI, respectively. Viral shedding was detected from cloacal swabs of 5.26% to 73.68% of the inoculated chickens at 1, 3, 5, 7, and 9 DPI, respectively. No viral shedding was detected from cloacal swabs of the inoculated chickens at 11 and 13 DPI (Figure 2B).

The results demonstrated that the novel triple-reassortant H6N8 virus not only replicated in multiple organs but also shed both in the respiratory system and the digestive system. The research provided more knowledge on the infectiousness and transmission potential of this substyle avian influenza virus.

### 3.3. Transmission of the Virus in Chickens

To investigate the transmissibility of the novel triple-reassortant H6N8 virus, five chickens were placed in the inoculated group to allow for contact with the inoculated chickens for 24 h post-inoculation. No obvious clinical signs were seen in all contacted chickens during the observation period. No viral shedding was detected from the oropharyngeal swabs of contacted chickens 1, 11, and 13 DPI. All contacted chickens shed the virus at 5 and 7 DPI. Viral shedding was detected from oropharyngeal swabs in 60% and 20% of the contacted chickens at 3 and 9 DPI, respectively. No viral shedding was detected from cloacal swabs in any of the contacted chickens at 1, 3, 11, or 13 DPI. Viral shedding was detected from cloacal swabs in 40% and 20% of the contacted chickens at 5 and 9 DPI, respectively. Peak viral shedding was observed on 7 DPI, with virus detection confirmed in all chickens (Figure 2B). The results suggested that the novel triple-reassortant H6N8 virus could be transmitted through the respiratory system and the digestive system of the inoculated chickens to contacted chickens.

### 3.4. Serological Analysis of Serum Samples from Chickens

We examined the humoral immune response in inoculated chickens and contacted chickens during the observation period. All of the serum samples were analyzed by HI test (Figure 2C). In the JM642-inoculated group, compared to 3 DPI, the average HI antibody titers were 2^4.5^ (*p* < 0.01), 2^7.9^ (*p* < 0.01), 2^8.2^ (*p* < 0.01), and 2^8.7^ (*p* < 0.01) at 5, 8, 11, and 14 DPI, respectively. For the chickens in contact with the JM642-inoculated group, the HI antibody could be not detected at 3 and 5 DPI. The HI antibody levels of these contact chickens increased dramatically over the next few days, and the average HI antibody titers reached up to 2^7.2^ (*p* < 0.01), 2^7.6^ (*p* < 0.01), and 2^8.4^ (*p* < 0.01) at 8, 11, and 17 DPI (Figure 2C). The results suggested that the novel triple-reassortant H6N8 virus replicated well in chickens, and a rapid and robust humoral immune response likely protected the host against disease, which was consistent with the findings we previously described about the replication, viral shedding, and transmission of the virus to chickens in this study.

### 3.5. Pathogenicity of the Virus to Mice

The mice, inoculated with a high dose of 10^8.0^ EID_50_, 10^7.0^ EID_50_, and 10^6.0^ EID_50_ of the virus, showed obvious signs of illness, including decreased activity, huddling, ruffled fur, and hunched posture. The mice began to die from 3 DPI. The mice with the dose of 10^5.0^ EID_50_ showed obvious weight loss from 6 DPI and started to recover and increase in body weight from 12 DPI to the end of the experiment (Figure 2D), and all mice survived the observation period. The JM642 virus had an MLD_50_ of 10^5.53^ EID_50_ (Figure 2E) and was medium pathogenic to mice based on 10^3^ EID_50_ < MLD_50_ values ≦ 10^6.5^ EID_50_ [25].

The novel triple-reassortant H6N8 virus replicated well in both the lungs and nasal turbinates of mice without prior adaptation (Figure 2F), with titers of 6.17 ± 0.14, 7.17 ± 0.14, 3.75 ± 0.6, and 4.25 ± 0.90 log_10_EID_50_/0.1 mL at 3 and 5 DPI, respectively. The virus replicated poorly in the hearts, livers, spleens, and brains, with titers ranging from 1.08 ± 0.14 log_10_EID_50_/0.1 mL to 3.08 ± 0.29 log_10_EID_50_/0.1 mL. Our findings indicate that the novel H6N8 virus is moderately pathogenic and causes systemic infection in mice. The ability of the virus to replicate efficiently in a mammalian model underscores its potential threat to public health and warrants continued surveillance.

## 4. Discussion

As the world’s largest poultry producer, China hosts a significant proportion of the global chicken and duck populations. The country’s diverse biosecurity measures and breeding models enable poultry farms and, in particular, live bird markets (LPMs), to function as critical reservoirs for various AIV subtypes and hotspots for genetic reassortment. This is especially true for low pathogenic avian influenza viruses (LPAIVs), which can circulate asymptomatically in domestic poultry. These environments also provide opportunities for close contact between humans and AIVs for the cross-species transmission of AIVs. Wu et al. reported the concurrent isolation of H7N9 and H9N2 viruses from a poultry farm, with reassortment between them generating five genotypes, one of which was responsible for human infection [26]. Surveillance data further indicated a considerable seroprevalence and seroincidence of AIV infection among the poultry workers, and multiple AIV subtypes (including H3N8, H5N1, H5N6, H5N8, H6N6, H7N9, and H9N2) co-circulated in the LPMs [27,28,29]. This co-circulation facilitates extensive gene segment reassortment both within and across subtypes and species. Thus, understanding the prevalence state of AIVs in poultry farms and LPMs is crucial for improving AIV monitoring systems in poultry and controlling the disease. In this study, phylogenetic analysis revealed that the novel H6N8 virus isolated from a chicken farm was a triple-reassortant virus, possessing its HA gene from the Eurasian lineage, an NA gene of North American origin, and six internal genes from H9N2 AIVs. Notably, no other AIVs were isolated from chickens in this farm. The route of viral introduction into the poultry farm remained unclear, with potential sources including farm workers, the poultry trade, or wild birds, and so on. The concern is that such a virus, if introduced into LPMs, might provide genetic segments for the emergence of new recombinant viruses. The terrestrial H6N1-like viruses, along with co-circulating H9N2 viruses, might have been involved in the genesis of the pathogenic H5N1 influenza viruses in 1997 that caused substantial human infection [30]. Therefore, future epidemiological surveillance of AIV should adopt a comprehensive strategy that encompasses not only poultry populations but also high-risk human contacts (e.g., farm workers, LPMs) and relevant environmental factors.

Among diverse subtypes of AIVs, H6 viruses are particularly notable for their high prevalence in both wild and domestic avian species, as well as their successful establishment in terrestrial poultry. In recent years, the frequency of H6 avian influenza virus (AIV) transmission from wild birds to domestic poultry has been increasing. H6 AIVs have not only facilitated their own adaptation to mammalian hosts but also contributed genetic material to the emergence of novel reassortant AIVs. Surveillance data indicate that H6 viruses isolated in southern China between 1972 and 2013 exhibited a progressive increase in binding affinity to human-like receptors, and approximately 34% of H6 isolates were capable of dual binding to both avian and human receptors [31,32,33]. A critical prerequisite for AIVs to cross the species barrier is the compatibility between cellular receptor availability and viral receptor-binding specificity, which can be altered through mutations and/or reassortment. In this study, we evaluated the infectivity, pathogenicity, and transmissibility of this novel triple-reassortant H6N8 virus in chickens. The results demonstrated that the virus replicated efficiently in multiple organs, with effective transmission among co-housed chickens, suggesting that these phenotypic traits might be mediated by its specific molecular characteristics. Notably, A 14-aa deletion was identified in the stalk region of the neuraminidase (NA) protein of the virus. Such truncations, a recognized molecular marker for AIV adaptation to Galliformes, are known to confer biological advantages in chickens, including enhanced replication, pathogenicity, and transmissibility [20,34]. Moreover, studies have demonstrated that the internal genes derived from H9N2 AIVs might endow the virus with a better ability to transmit among chickens and extend the host range of the novel avian influenza viruses. Recently, several new zoonotic influenza viruses have had emergence and spillovers to humans with their internal gene cassettes derived from enzootic H9N2 viruses, such as the H3N8/2022, H7N9/2013, H10N8/2014, and H10N3/2021 viruses [35,36]. Our finding that the novel H6N8 virus acquired its internal genes from H9N2 AIVs highlights its potential for increased adaptability, possibly leading to an expanded host range and more efficient transmission. Therefore, the combination of a short-stalk NA and H9N2 internal genes presents a plausible genetic basis for the observed efficiency in viral replication and transmission in our chicken model. However, the specific contribution of each element to the overall phenotype warrants further investigation.

In mice, the H6N8 virus demonstrated the capacity to infect and cause lethal disease without prior adaptation. Efficient viral replication was observed in the lungs and nasal turbinates, followed by systemic dissemination. Molecular analysis provided insight into the potential mechanisms underlying this heightened pathogenicity in mammals. While the HA protein retained the avian-type residues Q226 and G228, key mutations were identified in the receptor-binding site, including T155I, G158N, T160A, and E190V, which have been previously linked to enhanced binding to human-type receptors. Intriguingly, the HA also contained a lysine at position 137 (137K), a residue observed in 42% of H6 strains analyzed by Ni et al. [37]. Their study proposed that the triad N137, V190, and S228 in Taiwanese H6 HA may reduce the dependence on a hydrophobic residue at 226 and confer the virus with a slight preference for human receptors [37]. The functional role of 137K in receptor adaptation in our isolate, however, requires further investigation. Additionally, the NA stalk truncation has also been confirmed to enhance the pathogenicity of the AIVs in mice and ferrets [34]. Furthermore, several other mutations known to influence mammalian adaptation were also detected, such as PB2-G158E, PB2-I292V, PB2-A588V, PB1-M317V, PB1-P598L, PA-A515T, M1-N30D, and NS1-P42S. Collectively, these findings suggested that both adaptive mutations and reassortment events might contribute to the altered pathogenicity and host adaptation of the novel H6N8 virus in mice.

## 5. Conclusions

The novel H6N8 virus isolated from a chicken farm in southern China was identified as a triple-reassortant virus. It contains the HA gene derived from the Eurasian lineage, the NA gene from North American origin, and six internal genes originating from H9N2 AIVs. The virus exhibits adaptation markers acquired through substitution mutations and reassortment events, leading to enhanced infectivity and horizontal transmission in chickens and lethal infection in mice. These findings underscore the importance of genomic surveillance and the implementation of effective control measures against LPAIV infections in China.

## Figures and Tables

**Table 1:** Molecular characteristics of surface glycoproteins of the novel H6N8 virus.

Virus	HA																	NA		
	Receptor-Binding Sites ^a^										Potential Glycosylation Sites ^b^						Cleavage Site ^b^	Stalk Region ^c^	Drug Resistance Site ^d^	
	137	155	158	160	183	186	190	225	226	228	10–12	11–13	23–25	167–169	291–293	296–298	324–330	64–67	274	292
JM642	K	I	N	A	H	P	V	G	Q	G	NNS	NST	NVT	NNT	NKT	NVS	PQIETR↓G	D	H	R

^a^ all positions are in H3 number without signal peptide; ^b^ all positions are in H6 number without signal peptide; ^c^ all positions in N8 number; ^d^ all positions in N2 number.

**Table 2:** The changes in key amino acid sites in the internal genes of the novel H6N8 virus.

	PB2										PB1			PA			M1	M2					NS1	
	158	271	292	339	588	590	591	627	701	740	317	577	598	38	98	515	30	26	27	30	31	34	42	92
JM642	E	T	V	K	V	G	Q	E	D	D	V	K	L	I	T	T	D	L	V	A	N	G	S	D

## Data Availability

The original contributions presented in this study are included in the article. Further inquiries can be directed to the corresponding author.
